# Parapharyngeal and Retropharyngeal Abscesses in Children: A Report of Eight Cases

**DOI:** 10.3390/children12040487

**Published:** 2025-04-10

**Authors:** Matic Glavan, Lara Dreu, Boštjan Lanišnik

**Affiliations:** 1 Department of Otorhinolaryngology, Head and Neck Surgery, University Medical Centre Maribor, Ljubljanska Ulica 5, 2000 Maribor, Slovenia; matic.glavan@gmail.com; 2 Faculty of Medicine, University of Maribor, Taborska Ulica 8, 2000 Maribor, Slovenia; lara.dreu333@gmail.com

**Keywords:** deep neck space infections, parapharyngeal abscess, retropharyngeal abscess, pediatric airway management, neck abscess drainage, image-guided navigation

## Abstract

Background/Objectives: Deep neck space infections (DNSIs) in children, particularly parapharyngeal and retropharyngeal abscesses, pose a significant risk due to their ability to cause airway obstruction. The management of these infections in children differs from that in adults, requiring a multidisciplinary approach. This study aimed to evaluate the clinical presentation, diagnostic imaging, and surgical management of pediatric DNSIs. Methods: A retrospective review was conducted on pediatric patients (≤16 years) diagnosed with deep neck space infections between 2002 and 2022. A total of 266 cases were identified using ICD-10 codes, of which eight patients (3%) had para- or retropharyngeal abscesses. The clinical presentation, imaging modalities, airway management, and surgical approaches were analyzed. Results: Children with parapharyngeal or retropharyngeal abscesses had a mean age of 5.2 years, being significantly younger than those with peritonsillar abscesses (mean age: 13.5 years). The most common symptoms were a high fever (100%) and torticollis (63%). All patients underwent initial ultrasound (US), but a definitive diagnosis required contrast-enhanced CT or MRI. Seven patients (87.5%) underwent surgical drainage, with the decision to perform an incision dictated by the location of the abscess relative to vascular structures. Image-guided navigation facilitated minimally invasive transpharyngeal drainage in four cases. Postoperatively, six patients required prolonged intubation due to airway edema or surgical site management. One patient was successfully managed conservatively with antibiotics. Conclusions: DNSIs in children require prompt diagnosis and a tailored surgical approach. Imaging plays a crucial role in the localization of the abscess, with MRI preferred for detailed soft tissue assessment. Airway management is critical, and minimally invasive, image-guided techniques improve the precision of surgery. Multidisciplinary care optimizes patient outcomes.

## 1. Introduction

Deep neck space infections are potentially life-threatening conditions, especially in children, because they can cause airway obstruction. They are defined as infections between the fascial planes of the neck. There are 11 potential interfascial spaces between three layers of deep cervical fascia, and these are often classified into three groups based on their relation to the hyoid: suprahyoid (peritonsillar, submandibular, parapharyngeal, masticator/temporal, buccal, and parotid spaces), infrahyoid (pretracheal space), and the group that includes spaces along the entire length of the neck (retropharyngeal, prevertebral, danger, and carotid spaces) [1,2].

The parapharyngeal space is divided into the prestyloid and poststyloid compartments. The prestyloid compartment extends from the base of the skull to the superior cornu of the hyoid bone. It is bounded by the pharyngeal mucosal space medially and the superficial layer of the deep cervical fascia laterally. Posteriorly, it is bounded by both the carotid sheath and buccopharyngeal fascia (the middle layer of the deep cervical fascia). It contains fat, minor salivary glands, the internal maxillary artery, the ascending pharyngeal artery, and part of the pterygoid venous plexus. The retrostyloid compartment is basically the suprahyoid carotid compartment, containing the internal carotid artery and cranial nerves X, XI, and XII. The parapharyngeal space communicates with the retropharyngeal space that lies between the buccopharyngeal fascia, which covers the posterior pharynx and esophagus, and the alar fascia; it therefore occupies the space posterior to the pharynx and esophagus. It extends from the base of the skull down into the mediastinum to the level of the second thoracic vertebra. The retropharyngeal space is fused down the midline and contains two chains of lymph nodes that extend down each side [3].

Infection of the parapharyngeal or retropharyngeal space may spread in both directions. Retropharyngeal abscesses are unilateral as a result of the midline fusion. Retropharyngeal abscesses are primarily seen early in childhood because these lymph nodes tend to regress with age. They are most commonly caused by respiratory infections in children because these lymph nodes receive drainage from the nose, sinuses, and pharynx [3].

Other neck space infections, such as peritonsillar abscesses and lateral neck-level lymphadenitis with abscess formation, are more common, and their management is relatively straightforward in comparison to the management of para- and retropharyngeal abscesses. Deep neck abscesses in children are critical because they can cause airway obstruction and must therefore be addressed as an emergency. The management of parapharyngeal/retropharyngeal abscesses in children differs from that in adults [4].

The accurate recognition of these conditions is paramount for effective management. Imaging modalities such as sonography (US), computed tomography (CT), and magnetic resonance imaging (MRI) are essential in diagnosing deep neck abscesses in children. While US has limitations in its visualization of the retro- and parapharyngeal space compared to CT and MRI, the latter is typically restricted to general anesthesia, which may pose complications such as potential airway compromise during the diagnostic procedure. Additionally, the choice between CT and MRI is also influenced by considerations of radiation exposure and the use of iodine-based contrast in the former.

This case study of eight cases will address these differences, with an emphasis on airway management, dilemmas in diagnostic imaging, and surgical approaches, with a focus on minimally invasive approaches aided by image guidance.

## 2. Patients and Methods

We searched the hospital information system for patients up to the age of 16 who were admitted for a deep neck space infection from 2002 to 2022. We used the MCD 10 codes J36 (peritonsillar abscess) and J390 (retropharyngeal and parapharyngeal abscess) to identify patients of interest. The search returned 266 records matching the inclusion criteria. The documentation was reviewed, and it was found that 8 of the 266 patients (n = 8, 3.0%) had a para- or retropharyngeal abscess; meanwhile, the majority had a peritonsillar abscess (259, 97.0%). The clinical presentation, diagnostic imaging, multidisciplinary management, and outcomes were evaluated in a cohort of seven patients with a para- and retropharyngeal space.

## 3. Results

The mean age of the patients with a peritonsillar abscess (259 out of 266) was 13.5 years, but the mean age of the patients with a para- or retropharyngeal abscess was considerably lower, at 5.2 years.

The children with a parapharyngeal abscess most commonly presented with a high fever (100%, 8/8) and torticollis (63%, 5/8) at admission. All patients with a suspected deep neck abscess and/or lymphadenitis were referred to the Department of Otorhinolaryngology Head and Neck Surgery, where an initial clinical exam with neck sonography (US) was performed; a complete upper airway endoscopy was also performed to assess whether airway obstruction and swelling were present in order to obtain a diagnosis. Patients were admitted to the University Pediatric Clinic or the pediatric ICU, where standard blood tests such as CBC, CRP, procalcitonin, and biochemistry were performed. The blood samples were then sent for hemoculture and serology in order to detect EBV, toxoplasma, and Bartonella sp. Once an initial US and clinical exam of the neck had been performed, a multidisciplinary diagnostic and treatment plan was created, with a particular emphasis on airway management. The characteristics and presentation of the patients are presented in Table 1. Additional imaging was ordered in seven out of eight cases. CT and/or MRI with contrast was performed. CT and MRI imaging were performed under sedation, with the oversight of an anesthesiologist. Only in one case was a CT scan performed under general anesthesia with endotracheal intubation to avoid respiratory arrest. MRI was performed under general anesthesia with endotracheal intubation, with an airway team (pediatric ICU, anesthesiologist, and ENT) present during the procedure.

Table 2 summarizes the imaging results for the patients with suspected para- and retropharyngeal abscesses. The US results were similar in all cases, describing level II lymph nodes. Only in two cases was it possible to observe the collection of pus in the retropharyngeal space (patient 5) and parapharyngeal space (patient 6). MRI or CT scans were performed with contrast in both cases. CT was performed in four cases and MRI was performed in four cases. In one case, both modalities were used to diagnose a retropharyngeal abscess (patient 1). The US examination was able to detect the collection of pus in the lateral to carotid and jugular systems. In patient 6, US was used as the primary diagnostic method; this patient was intubated by a multidisciplinary airway team due to impending respiratory arrest. Once the first percutaneous incision had been made and the pus had been evacuated, CT with contrast was performed. Another collection superior, but still lateral, to the vessels was then performed. This collection was also drained using a separate percutaneous incision.

All patients received antibiotic therapy at admission; this usually comprised Amoxicillin with Clavulanate or Ceftriaxone with Clindamycin. The antibiotic therapy was modified according to the bacteria isolated. All bacteria isolated were sensitive to the first-choice antibiotics, but the therapy was modified with more narrow antibiotic coverage from empiric choice whenever possible: Amoxicillin with Clavulanate and Piperacilline/Tazobactam in patients 6–8, with Staphylococcus aureus isolated, were replaced with Flucloxacillin.

**Figure 1:**
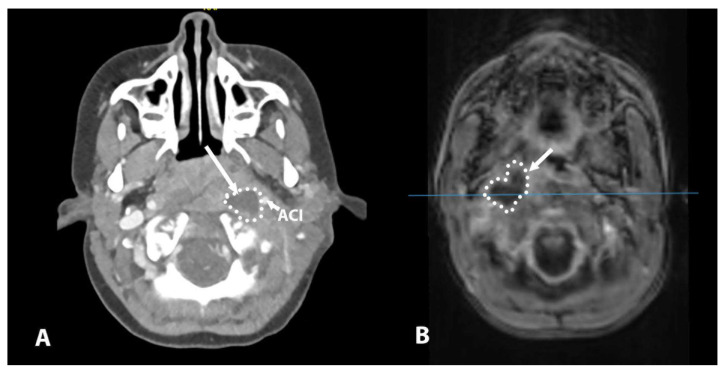
(**A**) CT scan showing a collection medial to the vascular structures (ACI) on the left side. (**B**) MR scan showing a collection medial to the vascular structures; note inferior image quality due to the child’s movement during the scanning procedure, despite sedation. In these cases, the route to the collection was achieved through the pharynx to avoid injury to the vessels. ACI—internal carotid artery.

**Figure 2:**
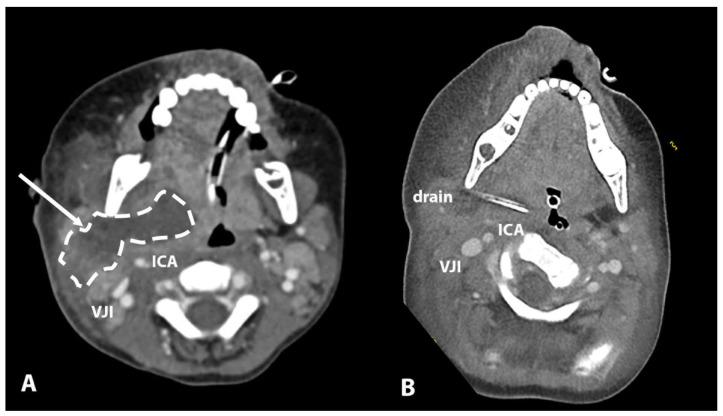
(**A**) Large collection pushing ACI and VJI in posterolateral direction. (**B**) Visualization of drain inserted through transcervical minimal incision. ICA—internal carotid artery, VJI—internal jugular vein.

**Figure 3:**
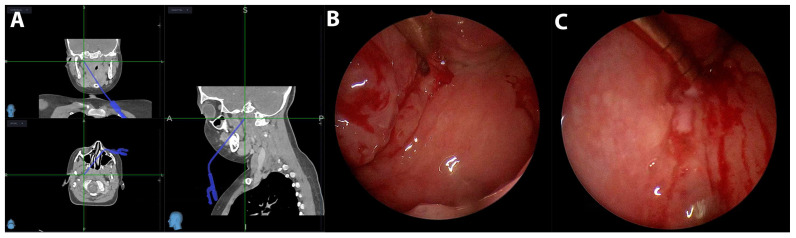
(**A**) A screenshot of image guidance in three planes and a suction tool guided to the collection. (**B**) Endoscopic view of the parapharyngeal incision below the Eustachian tube with suction inserted. (**C**) Endoscopic view of pus draining through the incision.

Seven out of the eight patients were treated surgically via a drainage incision. The surgical route to the pus collection was dictated by the position of the vascular structure, e.g., the internal carotid and jugular vein. When this collection was medial to the vascular structures, a transpharyngeal incision was created (Figure 1); however, if the collection was located lateral to the vascular structures, a transcutaneous incision was created (Figure 2). We explored the possibility to use a minimally invasive surgical approach; therefore, image guidance (Stealth ENT; Medtronic Inc. (Minneapolis, MN, USA)) was used to determine the best trajectory for the collection in four cases. Image guidance was used in three patients with a transpharyngeal approach under endoscopic control (Figure 3) and in one patient using the transcervical approach. In the transpharyngeal approach, the incision was placed below or at the level of the Eustachian tube; a navigated suction tool or probe was then inserted into the cavity of the abscess. In all three transpharyngeal incisions and the one transcervical incision, navigation helped to identify the collection on the first attempt (100% success rate).

In the other three patients with a retropharyngeal abscess and the one patient with a parapharyngeal abscess, no image guidance was used as the collection was clearly detectable in the clinical exam.

After surgical therapy (eight patients), the patients were either extubated (two patients, 25%) or transferred (six patients, 75%) to the pediatric ICU with an endotracheal tube in place. The patients were left intubated until no pus drained through the surgical incision and no dilatation was needed and/or until the upper airway edema subsided. The duration of the post-treatment intubation ranged from 1 to 6 days (average 3.2 days). On average, one or two dilatations were necessary (24 and 48 h after procedure), but the duration of intubation also depended on the presence of upper airway edema and the general condition of the child. An overview of the management and treatment of the patients is presented in Table 3.

## 4. Discussion

The surgical approach used to drain parapharyngeal and retropharyngeal abscesses in children should be carefully tailored according to the abscess’s location relative to the vascular structures. In this small series, we evaluated our experience and the literature regarding the management of para- and retropharyngeal abscesses. For abscesses positioned medial to the carotid artery and internal jugular vein, a transpharyngeal incision should be made. This technique facilitates direct drainage through the pharyngeal mucosa. In contrast, abscesses located lateral to the vascular structures necessitate a transcutaneous incision. The decision to pursue surgical drainage in most cases reflects the tendency for para- and retropharyngeal abscesses in children to rapidly progress, reducing the reliability of conservative management with antibiotics alone [5,6,7]. It is notoriously difficult to access deep neck collections in children, and a minimally invasive approach is preferred.

One of the primary causes of retropharyngeal and parapharyngeal abscesses in children is the spread of infections from the upper respiratory tract. Acute cervical lymphadenitis, often resulting from viral or bacterial infections, is frequently observed in young children, particularly those under six years of age. This demographic is particularly susceptible due to the high incidence of nasopharyngeal and oropharyngeal infections during early childhood. The Rouvière lymph nodes are particularly prominent in children and are known to regress by the age of 4 to 5 years. These nodes are responsible for draining the nasopharynx, oropharynx, and other structures in the head and neck, making them susceptible to infections that are caused by upper respiratory tract illnesses. Infections in these areas can lead to the suppuration of the lymph nodes, which may subsequently develop into retropharyngeal abscesses. Furthermore, studies indicate that the incidence of these abscesses is often associated with seasonal peaks in respiratory infections, particularly during the winter months, when acute tonsillitis is more prevalent [8,9].

In our study, the most common and reliable clinical sign of a para- or retropharyngeal abscess was the presence of a persistent fever, along with torticollis. A fever, sore throat, and torticollis are early symptoms of a deep space neck infection, whereas late symptoms include respiratory distress, hoarseness, neck stiffness, and stridor. The presence of cervical lymphadenopathy, or swollen lymph nodes, is another common finding; this occurs in a substantial percentage of cases [10,11].

Antibiotic therapy was initiated empirically and later tailored based on the microbiological findings, consistent with best practices [5,7].

The accurate diagnosis and localization of deep neck abscesses, including parapharyngeal and retropharyngeal abscesses, rely heavily on imaging modalities. While US has limited value in assessing deep neck infections, contrast-enhanced CT and MRI are crucial in determining the location and extent of deep neck abscesses [5,8,12].

CT is often the first-line imaging modality used in emergency settings due to its capacity for rapid acquisition and its high sensitivity during the detection of deep neck infections and abscesses. Studies indicate that CT has sensitivity of approximately 90% during the detection of neck abscesses, although its specificity is lower, at around 60%. This means that, while CT is effective in identifying the presence of an abscess, it may also misidentify other conditions, such as cellulitis or lymphadenopathy. The ability of CT to visualize bony structures and gas formation in soft tissues makes it particularly useful in cases where these factors are of concern. However, the radiation exposure associated with CT scans raises concerns, especially in pediatric populations, where minimizing radiation exposure is paramount [13,14,15].

In contrast, MRI is increasingly recognized as the gold standard in evaluating neck abscesses, particularly due to its superior soft tissue contrast and lack of ionizing radiation. MRI has demonstrated excellent sensitivity and specificity in detecting neck abscesses, with some studies reporting diagnostic accuracy that surpasses that of CT in certain contexts. MRI is particularly advantageous in pediatric patients, as it avoids the risks associated with radiation exposure, although it does require sedation in many cases. Moreover, MRI’s ability to provide detailed images of soft tissue involvement is crucial in determining the extent of infection and guiding treatment decisions. For instance, MRI can effectively differentiate between abscesses that require surgical intervention and infections that may be managed conservatively. This distinction is vital, as the improper management of neck infections can lead to severe complications, including airway obstruction and mediastinitis [13,16,17].

In our study, US was found to be insufficiently reliable in diagnosing para- or retropharyngeal abscesses, despite being non-invasive and widely used in initial assessments. While it identified lymphadenitis in all cases, it failed to reliably detect pus collections, highlighting the need for advanced imaging modalities such as contrast-enhanced CT or MRI. Both CT and MRI enabled the superior localization and delineation of abscesses, which is crucial for surgical planning. MRI was preferred for cases that required detailed anatomical visualization, despite its longer acquisition time and the need for general anesthesia. At our institution, we consider US as the primary imaging modality for the diagnosis of lateral neck pathologies. However, if the findings do not align with the clinical presentation and a DNSI is suspected, we perform a CT or MRI scan.

CT imaging provides valuable information about the neck spaces involved, the size and location of the abscess, and its relationship with the surrounding structures, including the airway and vascular structures. This information is crucial in guiding the surgical approach and in cases where image guidance is used [6,12,18,19].

In some instances, both CT with contrast and MRI may be utilized. While CT with contrast remains the first-line imaging modality due to its speed, availability, and ability to evaluate the airway, MRI offers superior detail when assessing the involvement of soft tissues and potential intracranial extension. Regardless of the modality used, imaging should be performed with the support of anesthesia and appropriate airway management to mitigate to ensure that the airway is not compromised by the abscess [5,8,12,18,19,20].

Image guidance, such as CT-/MRI-based navigation systems, was used in optimizing the surgical incision trajectory and helping the surgeon to use a minimally invasive approach. By precisely guiding the incision and suction/dissection tool to the abscess site, this minimally invasive approach reduces the risk of injuring critical structures [5,8]. We used image guidance in four cases with a retropharyngeal abscess. Image guidance reliably (100% success rate) guided the instrument into the abscess cavity via a transpharyngeal incision, under endoscopic control. A dissection probe or a suction tool was used to drain the pus. The incision was dilated in the following 24 h; therefore, the patients were left intubated until the abscess had resolved, as repeated dilatation in the pediatric population without general anesthesia is impossible.

The management of DNSIs in children is distinguished by its emphasis on securing the airway. In this study, the airways of all patients were evaluated by a multidisciplinary team comprising otolaryngologists, pediatric intensivists, and anesthesiologists. Intubation under controlled conditions was performed in patients whose airways were significantly compromised, ensuring safety during imaging or the surgical intervention. Some patients remained intubated after the initial procedure due to several reasons: impending airway compromise and the repeated dilatation of the surgical access point. It is impossible to assume that the child will comply with the repeated dilatation of the surgical drainage access point in the subsequent 24 h. Extubation was performed when the patients met the following criteria: (1) no airway compromise; (2) negative dilatation of the surgical access point; and (3) decreasing inflammatory parameters (CRP, leukocytosis, procalcitonin). In this series, the duration of intubation was between 24 h and 6 days.

In this series, one patient was managed conservatively (patient 4); in this case, the MR scan showed inflammatory changes in the soft tissue and retropharyngeal space, with suspected central colliquation. The patient responded well to conservative antibiotic therapy, with the follow-up MR showing a significant reduction in inflammatory changes and no residual abscess. This patient reflects the drawbacks of using MRI with contrast enhancement for decision-making when retropharyngeal deep neck space inflammation is present. MR is much more sensitive to inflammatory changes in the surrounding soft tissue and may overestimate the need for surgical intervention in comparison to CT with contrast; however, it exhibits better positive predictive values during the detection of inflammation (0.98) and abscesses (0.95) compared to the figures reported for CT with contrast [14]. It is also crucial to consider the timing of imaging as a potential factor. Early CT imaging may not adequately identify the abscess collection during the initial stages of the disease. Consequently, patients with no detectable abscess may require clinical monitoring and repeated imaging. However, it is essential to acknowledge the potential drawbacks of repeated radiation exposure in CT and repeated sedation in MRI. Therefore, the selection of the most appropriate imaging modality for follow-up should be based on the specific clinical scenario and a multidisciplinary approach.

## 5. Conclusions

In summary, we present a report and analysis of eight cases and the management strategies for parapharyngeal and retropharyngeal abscesses in children. The main limitation of this case study is the small sample size. Surgical drainage with a minimally invasive approach is the main therapeutical option. In preoperative evaluation, both CT and MRI can be employed in the diagnosis of neck abscesses in children. CT is favored for its rapid assessment and ability to visualize bony structures, while MRI excels in its characterization of soft tissue and avoidance of radiation exposure. The choice between these modalities often depends on the clinical scenario. According to our experience, it has been shown that image guidance could be a valuable tool to locate and drain the collection in the retro- or parapharyngeal space using a minimally invasive approach, but this should be verified also in future reports.

## Figures and Tables

**Table 1:** Characteristics and clinical presentation of the patients. CT—computed tomography, US—ultrasound, MRI—magnetic resonance imaging.

Patient	Age	Abscess Location	Antibiotic at Admission	Symptom Duration (days)	Presentation at Admission	Primary and Ancillary Diagnostic Procedure	Relation to Vascular Space	Airway Obstruction
1	6 years	Retropharyngeal	Clindamycin	5	Edema of the retropharyngeal space, high fever, regional lymphadenitis, stiff neck	US, CT, MRI	Medial	No
2	7 years	Parapharyngeal	Flucloxacillin	4	High fever, lymphadenitis, torticollis	US, MRI	Medial	No
3	6 years	Parapharyngeal	None	5	High fever, neck swelling, torticollis	US, CT	Medial	No
4	9 months	Retropharyngeal	None	4	High fewer, swelling neck, torticollis	US, MRI	Medial	No
5	18 months	Retropharyngeal	None	4	High fever, stiff neck, torticollis	US, MRI	Medial	No
6	3 years	Parapharyngeal	Flucloxacillin	4	High fever, neck swelling	US, CT	Lateral	Yes
7	7 years	Parapharyngeal	None	4	High fever, neck swelling	US	Lateral	No
8	10 years	Retropharyngeal	None	2	High fever, torticollis	CT	Medial	No

**Table 2:** Imaging study findings and airway management during imaging. CT—computed tomography, US—ultrasound, MRI—magnetic resonance imaging, ICA—internal carotid artery.

Patient	Age (years)	Abscess Location	US	CT	MR	Airway Management During Imaging	Relation to Vascular Space	Conclusive Imaging Method
1	6 years	Retropharyngeal	Enlarged lymph nodes in level II, no vascular involvement, no pathology visible that could explain symptoms	Retropharyngeal collection 50 × 6 × 18, medial to ICA, C2-C6	Retropharyngeal collection 25 × 6 × 40, medial to ICA, location oropharynx	Awake	Medial	CT, MRI
2	7 years	Parapharyngeal	Enlarged lymph nodes in level II, no vascular involvement, no pathology visible that could explain symptoms	/	Parapharyngeal collection 27 × 22 × 35, anterior and medial to ICA	Awake	Medial	MRI
3	6 years	Parapharyngeal	Enlarged lymph nodes in level II, no vascular involvement, no pathology visible that could explain symptoms	Parapharyngeal collection, medial to ICA, nasopharynx, below ICA canal, 18 × 15 × 12	/	Awake	Medial	CT
4	9 months	Retropharyngeal	Enlarged lymph nodes in level II, no vascular involvement, no pathology visible that could explain symptoms	/	Retropharyngeal inflammatory tissue 27 × 15 × 45 mm, medial to ICA with suspected central necrosis 11 × 6 × 27 mm	Sedation	Medial	MRI
5	1.5 years	Retropharyngeal	Enlarged lymph node level II at 35 mm, 20 mm (4 mL) of pus collection in retropharyngeal space, consistent with MR findings	/	Retropharyngeal collection 18 × 20 × 18 mm, medial to ICA, reaching up to clivus	Sedation	Medial	MRI
6	3 years	Parapharyngeal	Conglomerate of enlarged level II lymph nodes, area of collection in level II lateral to ICA	Major parapharyngeal collection 53 × 25 × 45, lateral and anterior to ICA	/	Intubation	Lateral	CT
7	7 years	Retropharyngeal	Enlarged lymph nodes level II (22 × 10, 20 × 8,10 × 10), no colliquation, vessels OK	Edema of the retropharyngeal and parapharyngeal space, hypodense area 16 × 11 medial to the ICA suspected of abscess	MRI not performed	Awake	Lateral	CT
8	10 years	Retropharyngeal	Enlarged level II lymph nodes, no vascular involvement, no pathology visible that could explain symptoms	28 × 11 × 11 mm retropharyngeal abscess medial to ICA and IJV	/	Awake	Medial	CT

**Table 3:** Overview of management and treatment of the patients.

Patient	Age	Abscess Location	Relation to Vascular Space	Airway Management After Therapy	Treatment	Image Guidance	Microbiology	Antibiotic Inpatient	Intubation Duration (days)
1	6 years	Retropharyngeal	Medial	Intubation	Transpharyngeal incision	-	*S. intermedius*, *Achromobacter* sp.	Clindamycin, Gentamycin, Ceftriaxone, Piperacillin	6
2	7 years	Parapharyngeal	Medial	Intubation	Transpharyngeal incision	Image guidance	*H. aphrophilus*	Clindamycin	2
3	6 years	Parapharyngeal	Medial	Intubation	Transpharyngeal incision	Image guidance	*S. pyogenes*, *S. oralis*	Clindamycin, Ceftriaxone	2
4	9 months	Retropharyngeal	Medial	None	Conservative	-	-	Clindamycin, Ceftriaxone	-
5	18 months	Retropharyngeal	Medial	Intubation	Transpharyngeal incision	-	*S. parasanguinis*, *S. oralis*, *S. vestibularis*	Amoxicillin/Clavunate	4
6	3 years	Parapharyngeal	Lateral	Intubation	Transcervical incision	Image guidance	*Staph. aureus*	Piperacillin/Tazobactam	4
7	7 years	Parapharyngeal	Lateral	None	Transcervical incision	-	*Staph. aureus*	Amoxicillin/Clavunate	-
8	10 years	Retropharyngeal	Medial	None	Transpharyngeal incision	Image guidance	*Staph. aureus*	Amoxicillin/Clavunate	1

## Data Availability

Data are not available for public use due to GDPR (General Data Protection Regulation EU 2016/6790) restrictions.

## Abbreviations

The following abbreviations are used in this manuscript:

DNSIs	Deep neck space infections
MR	Magnetic resonance imaging
CT	Computed tomography
US	Ultrasound
ICU	Intensive care unit
CBC	Complete blood count
